# The Effects of Different Femoral Component Materials on Bone and Implant Response in Total Knee Arthroplasty: A Finite Element Analysis

**DOI:** 10.3390/ma16165605

**Published:** 2023-08-13

**Authors:** Allegra Galas, Lorenzo Banci, Bernardo Innocenti

**Affiliations:** 1 LaBS, Department of Chemistry, Materials and Chemical Engineering “G. Natta”, Politecnico di Milano, 20133 Milan, Italy; 2 Clinical Department, Permedica Orthopaedics, 23807 Merate, Italy; lorenzo.banci@permedica.it; 3 BEAMS Department (Bio Electro and Mechanical Systems), École Polytechnique de Bruxelles, Université Libre de Bruxelles, 1050 Bruxelles, Belgium

**Keywords:** 3D printing, highly porous lattice, titanium alloy, CoCr alloy, press-fit, knee femoral component, stress shielding, finite element analysis

## Abstract

Due to the high stiffness of the biomaterials used in total knee arthroplasty, stress shielding can lead to decreased periprosthetic bone mineral density and bone resorption. As different materials and 3D-printed highly porous surfaces are available for knee femoral components from the industry nowadays, this study aimed to compare the effects of two same-design cruciate-retaining femoral components, made with CoCr and titanium alloy, respectively, on periprosthetic bone stresses through a finite element model of the implanted knee in order to evaluate the induced stress shielding. Moreover, the effect of the cementless highly porous surface of the titanium implant was analyzed in comparison to the cemented interface of the CoCr implant. The von Mises stresses were analyzed in different periprosthetic regions of interest of the femur with different configurations and knee flexion angles. The titanium component induced higher bone stresses in comparison with the CoCr component, mostly in the medial compartment at higher knee flexion angles; therefore, the CoCr component led to more stress shielding. The model was revealed to be effective in describing the effects of different femoral component materials on bone stress, highlighting how a cementless, highly porous titanium femoral component might lead to less stress shielding in comparison to a cemented CoCr implant with significant clinical relevance and reduced bone resorption after total knee arthroplasty.

## 1. Introduction

Total knee arthroplasty (TKA) has proven to be an effective surgical procedure with considerable clinical benefits in terms of quality of life, pain relief and knee functional restoration. The Australian Joint Replacement Registry 2022 Annual Report showed successful clinical outcomes in 90% of the patients with a 95.3% survival rate at the 10-year follow-ups [1].

Despite excellent long-term implant survivorship and significantly improved clinical outcomes, the major reason for late failure of a TKA, apart from periprosthetic infection, remains implant aseptic loosening, with 22% a distribution rate among all causes of failure [1,2]. Concurrent causes for late aseptic loosening are periprosthetic bone resorption due to implant-related bone stress shielding and wear-related periprosthetic osteolysis.

Design features, such as the material properties, geometry of the implant and fixation technique, are known to influence stress shielding and how the stress is transmitted to the underlying bone [3,4]. Stress shielding is a well-known phenomenon in primary TKA, which leads to a decrease in periprosthetic bone mineral density (BMD) secondary to a decrease in stress that the bone commonly receives [5].

Stress shielding occurs when there is a mismatch of the elasticity (Young’s modulus) between the implant’s metallic material and the surrounding bone, so that after surgery, the prosthesis, due to its significantly higher stiffness, carries a part of the load that was previously supported by the native bone, changing the mechanical environment of the periprosthetic bone [6]. The prosthesis shields certain areas of the bone from the physiological mechanical stimuli that are essential for its regular maintenance and remodeling, resulting in bone resorption in accordance with Wolff’s Law [7].

Consequently, to reduce stress shielding and postoperative bone resorption, it has been suggested that TKA components should be manufactured with materials that are less stiff, which are theoretically close to the native bone, to transfer forces similar to physiological conditions in order to maintain, as much as possible, the preoperative bone mass and structure. 

Many research studies have evaluated the effects of different knee design features on stress shielding through cadaveric experiments [8] or dual-energy X-ray absorptiometry (DEXA) to assess the amount of bone loss over time [3,5,9,10,11]. On average, a 15% decrease in BMD has been reported around the femoral component up to 24 months after TKA [12]. 

However, even if some authors evaluated the effects of component materials on stress shielding and subsequent bone resorption via finite element analysis (FEA), they mainly addressed the tibial side rather than the femoral side [6,13,14]. No studies regarding the effects of the materials of the femoral component on femur stress have been performed to the best of our knowledge. 

FEA can determine the internal bone stresses and allows simulations of several configurations, thus offering the possibility of determining the effect of the different materials on the same bone geometry, which cannot be investigated in vivo or in cadaveric bones [15,16,17]. 

The present study aims to analyze the periprosthetic stress transferred to the femoral bone around a new cementless, highly porous 3D-printed titanium (Ti6Al4V) knee femoral component compared to a conventional cemented Cobalt–Chromium-molybdenum (CoCr) femoral component of the same design through FEA in order to evaluate the potential benefit of using titanium for reducing stress shielding.

## 2. Materials and Methods

The present study utilized a finite element model, derived from a model that was previously validated and published [18,19]. This approach was chosen for its ability to conduct accurate biomechanical analyses [20], enabling the assessment of various configurations’ impacts on the same bone geometries (a comparison that cannot be investigated in vivo nor in cadaveric bones) [15,16,19,21].

In order to evaluate the influence of a new cementless 3D-printed titanium femoral component of a bicompartmental knee prosthesis on the distal femoral bone, the model was developed to analyze the bone stresses in the periprosthetic femur after the implantation and loading of the Ti6Al4V femoral component compared with the standard cemented CoCr component of the same design as a control. Since the CoCr alloy was stiffer than the Ti6Al4V alloy (E = 241 GPa versus E = 110 GPa, respectively), we hypothesized that the Ti6Al4V prosthetic component would be able to transfer more load to the femoral bone, and thus, the bone stresses would be higher than using the CoCr component.

Abaqus/CAE 2022 (Dassault Systèmes, Vélizy-Villacoublay, France) was used to develop the models and to perform all the finite element simulations. The model in this study included the following features.

### 2.1. Geometries

**Figure 1:**
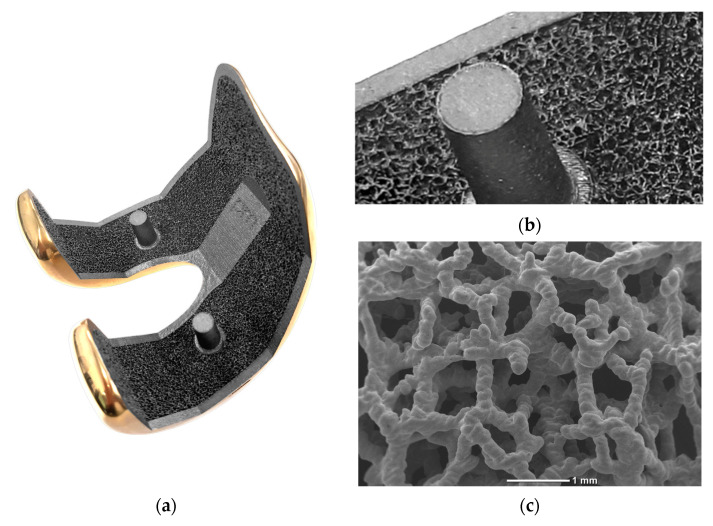
(**a**) The cementless press-fit 3D-printed Ti6Al4V femoral component featuring a ceramic layer coating of Titanium-Niobium Nitride (TiNbN) on the articular side and (**b**) featuring a highly porous lattice (Traser^®^) with a 1.35 mm thickness that was fully 3D-printed using selective laser melting without continuity solution with the solid portion of the component on the bone-facing side. (**c**) The highly porous lattice was a randomly irregular trabecular structure with 70% permeable porosity with a mean pore size of 520 microns, as seen at higher magnification in the SEM image. The titanium alloy had a low hardness and poor tribological performance due to the thin and unstable superficial passive oxide film, which was able to release particles. Thus, it required surface treatment, i.e., with a nitride-based ceramic coating through PVD, in order to be suitable for articular coupling against polyethylene [22].

The knee prostheses considered in this study were two cruciate-retaining (CR) fixed tibial platform femoral components, GKS Prime Flex (Permedica Orthopaedics, Merate, Italy), with the same design but with different bone fixations and manufactured with different materials and technologies. The investigational device was a cementless press-fit femoral component that was fully 3D-printed with selective laser melting with Ti6Al4V powder for additive manufacturing (ASTM F2924), which featured a randomly irregular, highly porous Ti6Al4V structure, commercially named Traser^®^, on the bone–side interface (Figure 1). 

This highly porous trabecular portion, 3D-printed in a continuous one-step process together with the bulky portion of the femoral component, had a depth of 1 mm, was included within the prosthetic component and had a further extra 0.35 mm of trabecular spikes protruding from the component surface to increase component’s press-fit and friction against the bone for optimal primary stability. This highly porous portion was developed to enhance the fixation of the femoral component through optimization of bone ingrowth within its fully interconnected porosity and bone osseointegration on the titanium trabeculae [23].

The femoral component used as a control was the cemented CoCr femur with exactly the same geometric design as the cementless, highly porous investigational femur.

The three-dimensional geometry of a physiological left femoral bone was obtained through reconstruction of Computer Tomography (CT) images of one intact fresh-frozen original left knee cadaveric specimen [18]. The reconstructed 3D CAD model of the femur was imported into the finite element software. The model had two distinct regions, which represented the cortical and the cancellous parts of the bone. This division enabled the two parts of the bone to have distinct mechanical properties, increasing the accuracy of the model. 

Since the main goal of this work was to assess the effects of different femoral component materials on the stress levels exerted on the femur, the ligaments were excluded from the model because they are not involved in the load transfer mechanism between the femur and the prosthesis. Their exclusion allowed us to reduce the computational cost without affecting the reliability of the model. For computational efficiency, the proximal portion of the femur was removed; a sensitivity analysis revealed that a longer bone length would have a negligible effect on periprosthetic bone stress distribution [6]. 

The two femoral components, together with the same UHMWPE-fixed CR insert, were virtually implanted into the femur according to the surgical guidelines provided by the manufacturer. According to the dimensions of the femoral bone, a size 10 was selected for the femoral component and a size FG with a thickness of 10 mm for the insert. 

### 2.2. Analyzed Configurations

A total of twenty configurations were examined in this study, considering the two materials for the femoral components (Ti6Al4V and CoCr), two bone properties (physiological and osteoporotic) and five knee flexion angles (0°–30°–60°–90°–120°).

The femoral components considered were particularly suitable for young and active patients since they were designed to allow a wide range of flexion and to optimize the performance of the prosthesis during flexion. For this reason, in the configurations studied, different knee flexion angles up to a maximum of 120° were simulated.

An additional configuration was analyzed to determine whether the difference in load transfer to the bone was due solely to the material of the femoral component (CoCr or titanium) or also due in part to the presence of the cement layer of the CoCr component. For this purpose, a single simulation at 90° of flexion was performed with the physiological bone and the CoCr femoral component, simulating the two possible anchoring methods: cemented or press-fit. 

### 2.3. Material Models and Properties

Bone is an inhomogeneous material; however, the cortical and cancellous bone were considered homogeneous within the region where they were defined. The values of the bones’ mechanical properties were obtained from the literature (Table 1) [15,17,24,25].

There are different modeling methods of the mechanical behavior of bone tissue. In this study, in agreement with previous studies present in the literature [15,16,18,19,26], the cortical bone was modeled as a transversely isotropic material with properties varying according to the different axes. The third axis was taken parallel to the anatomical axis of the bone. The cancellous region of the femur was modeled as an isotropic material [15]. 

To model the osteoporotic bone, the Young’s modulus of the cortical bone was reduced by 32% while the Young’s modulus of the cancellous bone was reduced by 66%; the Poisson’s ratio remained constant for both healthy and osteoporotic bone qualities [15].

The two femoral components were made of a Cobalt–Chromium alloy (CoCr) and a titanium alloy for biomedical use (Ti6Al4V).

The porosity of the Traser^®^ trabecular structure was not included in the model, while its properties were taken from experiments on trabecular specimens with 70% porosity [27].

The CoCr femoral component was fixed to the bone by applying a cement layer with a constant thickness of 3 mm over the resected surface of the femur. The material adopted for the cement was PoliMetilMetAcrilate (PMMA). For the polymeric insert, Ultra-High-Molecular-Weight Polyethylene (UHMWPE) was employed. All the materials were assumed to be homogeneous, isotropic and linear elastic [15,16,19,20,26,28].

### 2.4. Load and Boundary Conditions

A compression force of 2600 N was statically applied along the anatomical femoral axis [18,29,30,31] and distributed over the distal surface of the tibial insert. This condition replicated the maximum knee axial force during gait, which corresponds to about 3.2 times an 80 kg body weight [18,31].

The tibial insert was constrained in all directions except for the displacement along the vertical axis and the rotation around it, while the femur was completely fixed at its proximal end with an encastre [15].

As our objective was to examine the specific impact of material stiffness on periprosthetic bone stress, we maintained a consistent implant design, load and boundary conditions for each configuration. This approach enables us to obtain comparable results across the different configurations [15,20,21].

### 2.5. Finite Element Model Definition

**Figure 2:**
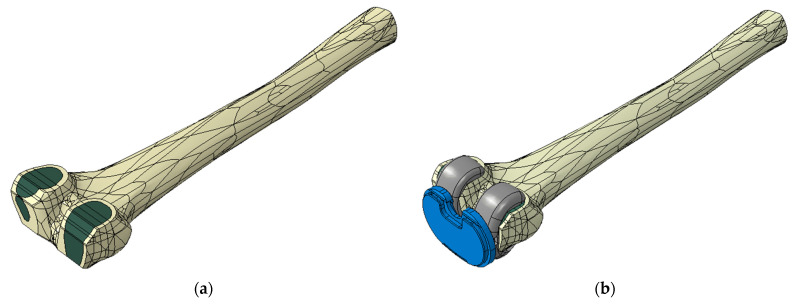
(**a**) Resected femur: cortical bone is shown in yellow and cancellous bone in green; (**b**) view of the complete finite element model with the femoral component (grey), the polymeric insert (blue) and the femur.

Two FE models were created, one for each femoral component’s material, and each model was composed of the resected femur, a femoral component and the tibial insert. The view of the complete model is shown in Figure 2b.

All the three-dimensional structures were meshed using four-node linear tetrahedron elements with sizes ranging from 1.8 to 3 mm.

**Figure 3:**
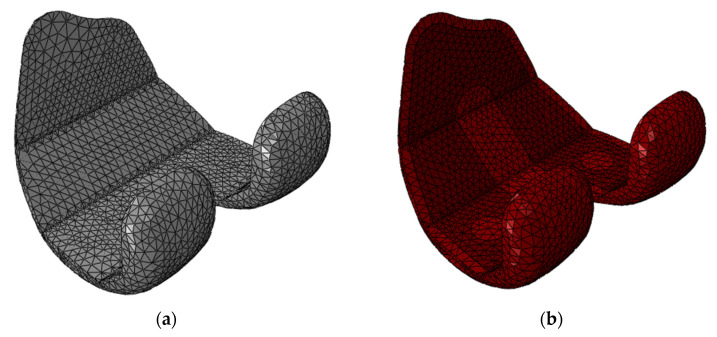
The two femoral components were meshed with 2.5 mm tetrahedron elements. (**a**) CoCr cemented femoral component; (**b**) Ti6Al4V cementless femoral component with the highly porous trabecular portion colored in dark red.

The total elements were 30,045 for the CoCr femoral component, 46,350 for the titanium femoral component, 46,126 for the insert and 194,487 for the femur (Figure 3). 

The interface between the anterior face of the resected femur and the anterior internal face of the femoral component was assumed to be tied.

All the interactions between the implant and bone or between implant components were modeled using a “surface to surface” contact formulation, with a friction coefficient that varied depending on the materials involved.

The coefficient of friction for the interactions between the femoral components and the tibial insert was set to *μ* = 0.05 for the CoCr–UHMWPE interface and *μ* = 0.2 for the Ti6Al4V–UHMWPE interface [22]. The coefficient of friction for the interactions between the cement layer and the bone was set to *μ* = 0.25, while for the Ti6Al4V–bone interface, a value equal to *μ* = 0.6 was used [22,24].

The interface between the cement layer and the CoCr femoral component was assumed to be fully bonded since the cement layer had been applied on the femoral component using the “skin” function.

Since in the software, the forces and the boundary conditions were applied to a single reference point (RP), a coupling interaction between it and the distal surface of the insert was necessary to apply the axial load and the boundary conditions to the tibial insert.

In order to determine the outputs of the simulations, some regions of interest (ROIs) had to be defined. These regions were identified in correspondence with the femur bone and the polymeric insert according to the main aim of this study.

**Figure 4:**
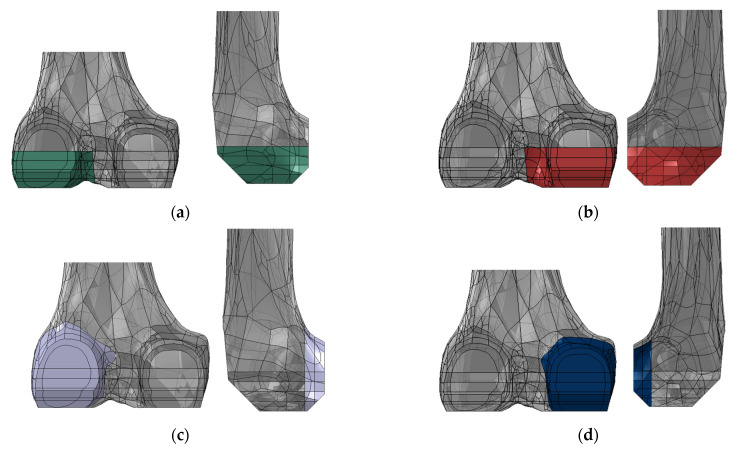
Posterior (on the left) and lateral views (on the right) of (**a**) the lateral distal ROI; (**b**) the medial distal ROI; (**c**) the lateral posterior ROI; and (**d**) the medial posterior ROI.

Four ROIs were identified in the femur by subdividing it into sections that were defined by partitioning the femur with planes parallel to the distal femoral cut and to the posterior femoral cut (Figure 4). The periprosthetic distal region of the femur was represented by two ROIs (the lateral distal ROI and the medial distal ROI), while the periprosthetic posterior region of the femur was represented by two other ROIs (the lateral posterior ROI and the medial posterior ROI). The distal ROIs included the first 20 mm of the distal femur, and the posterior ROIs included the first 10 mm of the posterior femur, while they spanned the entire mediolateral width.

In each ROI, cortical and cancellous sections were considered and average von Mises stresses were computed and compared among the different configurations.

In order to determine whether there was any difference in contact at the tibio–femoral interface due to changing the material of the femoral component, the contact forces and the contact areas between the tibial insert and the two types of femoral components were evaluated in correspondence with the proximal surfaces of the polymeric insert dividing it into two ROIs: the lateral one and the medial one. 

## 3. Results

### 3.1. Femur Average Stress

**Figure 5:**
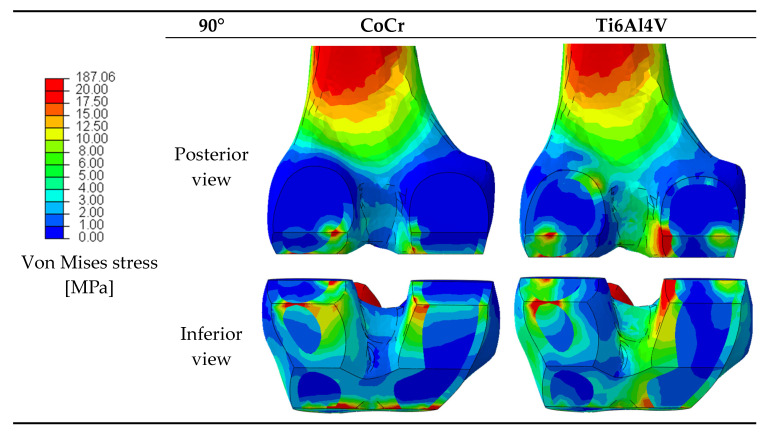
Posterior and inferior views of the von Mises stress distributions in the periprosthetic femur after the implantation of the two femoral components at 90° of flexion.

The material properties of prosthetic components can affect the stress distribution in the periprosthetic bone [32,33]. Since variations in the stress distribution may be responsible for bone resorption around the prosthesis as a result of stress shielding, the stresses in the femur after the implantation of the two femoral components were analyzed. The von Mises stresses were extracted and Figure 5 provides a graphical overview of the stress in the periprosthetic distal region of the femur for the considered materials at 90° of flexion. 

The average von Mises stresses were calculated to determine with which femoral component’s material the bone was more loaded and in which zone this occurred. The average von Mises stress in each ROI of the periprosthetic femur at 0°, 30°, 60°, 90° and 120° of flexion was extracted for all the configurations. The quantitative values of the average stresses are reported in Table 2.

Comparing the behavior of the two femoral components in the transmission of the load to the bone, we can say that at 0° of flexion, the CoCr femoral component transmitted slightly more load to the bone in the lateral distal ROI, while in the medial distal ROI, the behavior was the same between the two components.

At 30° of flexion, the CoCr component performed better at transferring more load to the bone in the lateral ROIs (lateral distal and lateral posterior) as opposed to the titanium component, which transmitted more load in the medial ROIs (medial distal and medial posterior). 

At 60° of flexion, with the Ti6Al4V component implanted, the bone was more loaded in all the ROIs except for the lateral distal, whereas at 90° and 120°, it performed better in all the regions of interest.

At low flexion angles (0° and 30°), the bone was almost equally loaded with the CoCr femoral component and with the Ti6Al4V one, whereas at higher degrees of flexion (60°, 90° and 120°), the Ti6Al4V component performed better and transferred more load to the bone than the CoCr one.

**Figure 6:**
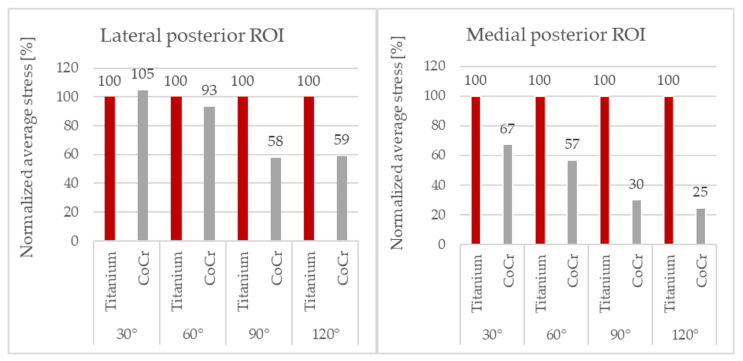
Normalized von Mises average stresses in the femur’s lateral posterior and medial posterior ROIs at 30°, 60°, 90° and 120° of flexion.

To be able to perform a clearer comparison, the values of the average von Mises bone stresses obtained with the CoCr component were normalized with respect to those obtained with the Ti6Al4V component implanted. As the posterior ROIs were more loaded than the distal ROIs during flexion, these were the areas of greatest interest and only these results are reported (Figure 6).

The average stress values with the Ti6Al4V component were generally higher than the those with the CoCr component, mainly in the medial compartment.

At 120° of flexion, the CoCr component showed a reduction in stress in the femur of up to 75% in the medial posterior ROI and 41% in the lateral posterior ROI compared to the titanium component.

A relationship between the difference in bone stress between the two femoral components and the degree of flexion could be observed: at higher degrees of flexion, the difference in load transfer became more evident and relevant, with the Ti6Al4V component transmitting more load to the bone in comparison to the CoCr component.

In fact, analyzing the percentage variation, at 120° of flexion in the medial posterior ROI (Figure 6), the difference reached its maximum, and the bone stress with the Ti6Al4V component was four times that of the CoCr component.

The results from the model with osteoporotic bone conditions supported the stress trend observed in the simulations with the physiological bone in a similar way (Table 2).

**Figure 7:**
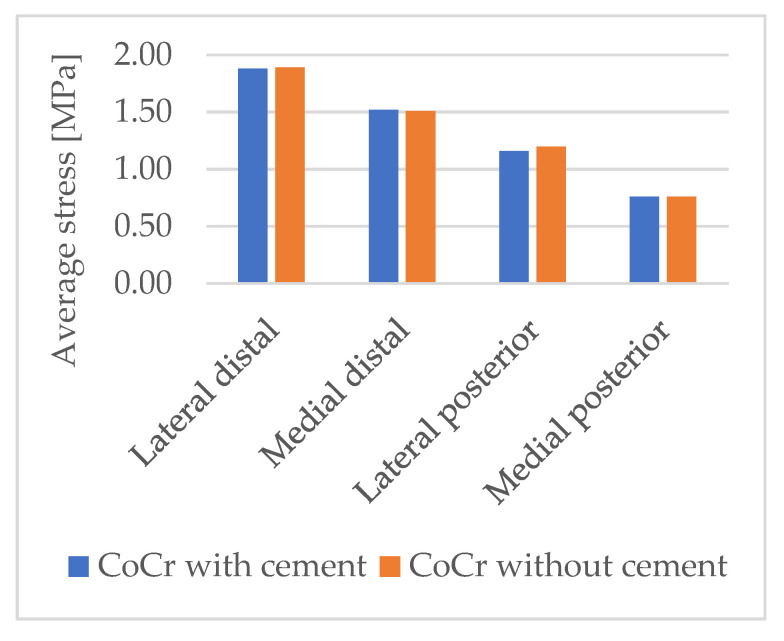
Magnitude of average von Mises stresses in the femur’s ROIs with the CoCr femoral component implanted at 90° of flexion, with and without the cement layer.

To determine whether the difference in load transfer to the bone was due solely to the material of the femoral component or also due in part to presence of the bone acrylic cement layer with the CoCr component, a single simulation at 90° of flexion was performed with the CoCr femoral component, simulating two possible anchoring methods: cemented or press-fit (Figure 7).

There was no relevant difference in the bone’s stress between the two cases; therefore, it can be concluded that any differences in the stress on the bone with the two implanted femoral components were only due to the material used for the femoral component and not the fixation technique of the CoCr component.

The average von Mises stresses in the two femoral components at 0°, 30°, 60°, 90° and 120° of flexion were also evaluated, and in none of the configurations analyzed were the stresses found to be above the mechanical strength limits of the materials they were made of.

### 3.2. Polymeric Insert Contact Area and Contact Force

The magnitude of the contact force acting on the polymeric insert as well as the contact area between the tibial insert and the femoral component at the tibio–femoral interface were measured on the proximal surface of the insert in two situations: with the Ti6Al4V femoral component implanted and with the CoCr one.

This result ensures that, whenever the component material changes while maintaining the same design, the tibial–femoral contact areas and forces do not change along with the material.

In all the configurations, the contact area and contact force with the CoCr component were similar to the ones with the Ti6Al4V component, and no substantial differences were reported.

## 4. Discussion

The aim of this study was to quantify the change in femoral bone stresses in the knee induced by the use of two femoral components of the same design, but made with different materials and fixation, in order to verify the hypothesis that an implant with a lower modulus of elasticity, such as Ti6Al4V, could allow better stress transmission in terms of higher stresses in the periprosthetic bone in comparison to a CoCr implant that is characterized by stress shielding.

The material properties of the prosthetic components affect how the load is transferred to the bone. A material that is stiffer than the native bone, such as metal alloys, leads to stress shielding, a mechanical phenomenon in which the forces that were entirely transmitted through the bone in physiological conditions are more supported by the implant itself rather than by the bone after implantation.

In our study, we found that the cementless Ti6Al4V femoral component performed better in almost all the regions of interest, mostly at higher degrees of knee flexion (60°, 90° and 120°), showing more load transfer from the prosthesis to the bone than the CoCr one, especially in the medial femoral condyle. In fact, at 120° of flexion, the CoCr component showed a reduction in stress in the femur of up to 75% in the medial posterior ROI and 41% in the lateral posterior ROI compared to the titanium component. We observed how the lower stiffness of the Ti6Al4V femoral component permitted higher load transfer to the bone than the CoCr component did, because the elastic modulus of the Ti6Al4V alloy was almost half of the elastic modulus of the CoCr alloy, and a reduction in Young’s modulus corresponded to a decrease in the stress-shielding effect in the periprosthetic femur.

Previously, the effects on distal femur stresses caused by different knee femoral component materials have been investigated by few studies. In 2013, the effects of a designed knee femoral component with functional graded biomaterials on distal femur stresses were investigated using a three-dimensional finite element modeling, showing an increase in bone stress of up to 41% with the knee in full extension in comparison with a femoral component with standard material [34,35]. A more recent study found increased strain energy density, relative to the preoperative bone, with a new poly-ether-ether-ketone (PEEK) knee femoral implant compared to a standard CoCr implant [36]. All these studies showed how a less-stiff femoral component is able to induce higher stresses on the femoral bone as we found in our study. There is also a paucity of finite element model studies on bone stresses regarding knee tibial components. Some finite element modeling studies have found higher tibial bone stresses with porous tibial components [37,38]. Other studies have shown that differences in tibial platform design result in different stresses on the bone [39].

Porous prosthetic designs seem to be promising to reduce stress shielding since the introduction of porosity into implant materials, facilitated by modern additive manufacturing technologies, has been proven to enable significant implant stiffness reduction [40]. Hence, introducing porosity into implant materials has a dual effect: it mitigates stress shielding by reducing implant rigidity and, at the same time, enhances implant fixation.

The effects of different prosthetic design features on load transfer to the periprosthetic bone have mainly been assessed throughout long-term follow-up clinical studies with comparative radiographic and DEXA scan analyses to determine the amount of periprosthetic bone mineral density changes in patients over time after TKA [3,5,9,10,11].

Cemented CoCr tibial trays, as well as thicker CoCr tibial trays, have been found to cause bone resorption, mainly in the medial side of the tibia, through radiographic measurements of bone margin remodeling [5,11]. Comparing cemented vs. cementless TKA, the existing literature has not discovered any differences in stress shielding on the tibial side [3,10]. However, on the femoral side, significant differences in the BMD measured by DEXA scans were found between the DePuy Attune cemented and press-fit in two out of three regions of interest of the femoral zones evaluated, with a significantly higher BMD with the press-fit femoral component [3]. However, uncemented press-fit femoral components have been shown to lead to significantly lower BMD values over 2 years of follow-up after TKA in comparison to preoperative values [40,41].

The increased stresses found in our finite element model in the periprosthetic femoral bone around the Ti6Al4V femoral component might have a significant clinical implication in total knee arthroplasty. According to Wolff’s Law, the increased stresses transferred to the femoral condyles, mainly in the posterior condyles, reducing stress shielding, might lead to less periprosthetic bone resorption over time after implantation in comparison to a standard cementless press-fit CoCr femoral component. Moreover, this theoretical reduction in bone resorption could involve the posterior femoral condyles, where bone defects are usually most frequently found during revision of TKA, as in AORI type IIa-b defects [42]. Thus, this result could have an important role for the mitigation of postoperative periprosthetic fracture and implant aseptic loosening risks, as well as having the possibility for easier subsequent revision surgery due to the higher quality and quantity of the residual femoral bone stock. 

In order to verify if the fixation method could have an impact on stress shielding, in our model, a simulation was performed with the CoCr femoral component, modeling the two possible fixation methods to determine whether the difference in load transfer to the bone was attributable only to the material of the femoral component or also in part to the fixation method. The results of this simulation confirmed the findings from the literature that there is no difference in the load transfer between the two different fixation methods. Therefore, it can be concluded that any differences in the stress on the bone between the two implanted femoral components were solely attributable to the femoral component’s material and not to the CoCr component’s fixation method.

The results of the present study have been extracted and evaluated, while keeping in consideration the information coming from the literature, in order to give a biomechanical overview of the effects of the different materials addressed.

Our findings support the previous study of Yoon, which evaluated stress shielding and consequent periprosthetic bone resorption with radiolucent lines through analyzing the changes in bone mineral density rather than with finite element analysis [43].

Martin studied the effects of tibial component materials on stress shielding through a radiographic comparative analysis [11]. Both Yoon and Martin reported a greater degree of stress shielding with CoCr implants than with Ti6Al4V implants. Even though these studies are based on tibial implant material rather than femoral component materials, we obtained similar results in our computational comparative study.

Our findings led us to believe that the stiffer CoCr femoral component increases femoral stress shielding, which results in decreased bone stress compared to the Ti6Al4V femoral component. The results of the finite element analysis prove that, as expected, the change of material of the femoral component has no impact on the contact area or on the magnitude of the contact force at the tibial–femoral interface. Consequently, it can be concluded that there is no dependency between the femoral component material and the contact area and contact force if the design is maintained.

As important catastrophic failures of some modern porous tibial baseplate designs were reported in the literature [44,45], an assessment of the von Mises stresses of the two femoral components was conducted under various flexion angles. Nevertheless, none of the analyzed configurations exhibited stresses surpassing the mechanical strength thresholds of the respective materials.

Our study has several limitations. First, the bone femur with the implanted femoral component models was loaded with a static compressive force equal to the maximum value of load during normal gait. Thus, the model did not address more physiological loading conditions. Secondly, we considered a limited number of femoral ROIs: medial distal, lateral distal, medial posterior and lateral posterior. No ROI was considered in the anterior portion of the femur behind the prosthetic trochlear shield as De Ruiter and Yilmaz considered in their work [36,46]. De Ruiter et al. found higher stress loading in the anterior femoral region with a PEEK knee femoral component in their FEM study, while Yilmaz et al. found a significant BMD reduction, mainly in the anterior femur region, after TKA. Therefore, important information on the anterior femur region was missing in this study. Thirdly, we used only one finite element model, developed from one cadaveric femur, instead of more models from several different femurs. This limitation did not enable our study to use statistical analysis and did not allow us to take the anatomical diversity of femurs between patients into consideration. Lastly, we considered only a single implant position, and we did not consider other possible positioning or malpositioning, but this was beyond the scope of our study.

Future research could involve analysis on the effects of different femoral component materials through a dynamic analysis with cyclic loadings over time. Another development of the model may also concern the implementation and the integration in the finite element simulations of a strain-adaptive bone remodeling algorithm capable of predicting bone remodeling. This model could be considered reliable in the indication of a trend regarding the effects of implant materials on bone response, even if further studies are required to define the effective reliability and accuracy of the results. To provide physiologically valid predictions, the model should be validated with in vitro and clinical results.

Therefore, the results of this study have to be considered as a comparison of the different configurations and, thus, mainly have a comparative rather than an absolute value. It is, however, to be reported that, despite the limitations listed above, the outcomes of the simulations found are in agreement with experimental, numerical and clinical results available in the literature and support their relative discussions and conclusions.

## 5. Conclusions

The purpose of this finite element analysis study was to determine the bone stress distribution transferred to the distal femur of the knee resulting from implanted models of a new cementless, highly porous Ti6Al4V femoral component in comparison to the same design in a cemented CoCr version used as a standard comparator device. 

The results obtained from the analysis confirmed the hypothesis that the distal femoral bone might receive higher stresses, mainly distributed in the posterior portion of the femoral condyles, when loaded in knee flexion conditions with an implanted cementless, highly porous femoral component of lower stiffness made of Ti6Al4V alloy compared to CoCr. In conclusion, these results, although limited to a computational knee implant model simulation, highlight the fact that Ti6Al4V alloy as an implant material for knee femoral components might reduce the stress shielding phenomenon in the knee after TKA, leading to less bone resorption in the femoral condyles, thus manifesting in beneficial effects for the patients.

The present study can be considered as a rationale for further clinical studies aiming to investigate bone mineral density changes after TKA with this new cementless, highly porous femoral component.

## 6. Patents

A European patent application has been submitted regarding the investigational device.

## Figures and Tables

**Table 1:** Full overview of material’ properties in terms of Young’s modulus and Poisson’s ratio [15,18,24].

	Young’s Modulus E [MPa]			Poisson’s Ratio ν [–]		
	E_1_	E_2_	E_3_	ν_12_	ν_13_	ν_23_
**Cortical bone**	11,500	11,500	17,000	0.58	0.31	0.31
**Cortical osteoporotic bone**	7820	7820	11,560	0.58	0.31	0.31
**Cancellous bone**	2130			0.3		
**Cancellous osteoporotic bone**	724			0.3		
**CoCr (ISO 5832/4)**	241,000			0.3		
**Ti6Al4V (ASTM F2924)**	110,000			0.3		
**Traser** **^®^ Ti6Al4V** **(ASTM F2924)**	1440			0.35		
**UHMWPE (ISO 5834/2)**	920			0.44		
**PMMA**	3000			0.3		

**Table 2:** Von Mises average stresses in the femur for Ti6Al4V and CoCr femoral components according to the identified region of interest at each knee flexion angle considered. The von Mises stress values found with osteoporotic conditions are in parentheses.

Region of Interest	0°		30°		60°		90°		120°	
	Ti6Al4V	CoCr	Ti6Al4V	CoCr	Ti6Al4V	CoCr	Ti6Al4V	CoCr	Ti6Al4V	CoCr
Lateral distal	1.58 *(1.47)*	2.08 *(1.76)*	2.39 *(2.06)*	2.75 *(2.18)*	2.01 *(1.47)*	2.87 *(2.37)*	1.91 *(1.39)*	1.88 *(1.42)*	2.19 *(2.15)*	1.87 *(2.11)*
Medial distal	1.90 *(1.67)*	1.87 *(1.57)*	2.31 *(1.99)*	2.11 *(1.69)*	2.45 *(2.10)*	2.06 *(1.66)*	2.73 *(2.03)*	1.52 *(1.16)*	2.87 *(3.16)*	1.60 *(1.70)*
Lateral posterior	1.75 *(1.94)*	2.64 *(2.80)*	3.38 *(3.27)*	3.54 *(3.83)*	2.72 *(2.32)*	2.53 *(2.69)*	2.01 *(1.84)*	1.16 *(1.09)*	2.40 *(2.61)*	1.41 *(2.24)*
Medial posterior	2.20 *(2.40)*	2.34 *(2.39)*	3.50 *(3.78)*	2.36 *(2.61)*	2.96 *(3.09)*	1.68 *(1.87)*	2.53 *(2.10)*	0.76 *(0.73)*	2.93 *(3.62)*	0.72 *(1.23)*

## Data Availability

Research data supporting the reported results can be available upon direct request to the corresponding author.
